# INF-**α** and Ototoxicity

**DOI:** 10.1155/2013/295327

**Published:** 2013-07-25

**Authors:** Mohammad Reza Sharifian, Shima Kamandi, Hamid Reza Sima, Mohammad Ali Zaringhalam, Mehdi Bakhshaee

**Affiliations:** ^1^Otorhinolaryngology-Head & Neck Surgery Department, Imam Reza Hospital, Faculty of Medicine, Mashhad University of Medical Sciences, Mashhad 9137913316, Iran; ^2^Otorhinolaryngology-Head & Neck Surgery, Mashhad University of Medical Sciences, Mashhad, Iran; ^3^Gastroenterology Department, Imam Reza Hospital, Faculty of Medicine, Mashhad University of Medical Sciences, Mashhad 9137913316, Iran; ^4^Sinus and Surgical Endoscopic Research Center, School of Medicine, Mashhad University of Medical Sciences, Mashhad, Iran

## Abstract

*Introduction*. INF-**α** is a common drug for the treatment of hepatitis B and C. Although a variety of related complications are discussed, possible ototoxic effects of this mediation are not well described. *Methods and Materials*. In a before-after control study, 24 patients who received INF-**α** for the treatment of hepatitis B and C and 30 normal controls were included. Subjective and objective ototoxicity evaluations via questionnaire, high frequency audiometry, and measuring transiently evoked otoacoustic emissions (TEOAEs) were performed one week before and one month after the prescription of the drug. *Results*. Subjective hearing complaint, tinnitus, and vertigo were seen in just 3 cases, which was not statistically significant (*P* = 0.083). In the frequency range of 4000 to 8000 Hz before (9.38 ± 1.0 and 10.7 ± 1.2, resp.) and after (17.9 ± 2.6 and 17.6 ± 2.6, resp.) one month of treatment, a significant difference (*P* = 0.083) was detected. Progressive decreases in amplitude of the OAE during TEOAE measurement in 1, 2, and 4 frequencies among 41.66%, 18.75 %, and 43.75% were observed, respectively. The hearing loss was seen more among older and male cases significantly. Conclusion. The results showed ototoxicity of INF-**α** that may encourage planning hearing monitoring in patients receiving this drug.

## 1. Introduction

 The overall incidence of chronic hepatitis is 2%, with increased incidence in certain high-risk populations [1]. The traditional treatment for chronic hepatitis B is one course of standard interferon-*α* (INF-*α*). Pegylated-INF-*α* is widely used instead of standard INF-*α* because of its ease of use and superior results. INF-*α* is injected subcutaneously once per week, for 24 to 48 weeks. The most common complications of treatment include influenza-like symptoms (headache, fever, chills, myalgia, and weakness), which occur within 6 hours of beginning treatment in more than 30% of patients during the first week of treatment. Other potential complications include neurotoxicity (mood disorders, depression, confusion, convulsion, and drowsiness), bone marrow suppression, chronic fatigue syndrome, weight loss, skin rash, coughing, myalgia, alopecia, tinnitus, reversible hearing loss, retinopathy, pneumonia, and cardiac toxicity. An autoantibody phenomenon may also occur, which leads to flare-up of autoimmune diseases or subclinical thyroid disease [2]. 

Published papers on hearing loss and tinnitus due to treatment with INF-*α* are mostly case reports with some limited prospective studies. The first report of auditory complications associated with INF-*α* treatment was published in 1994 [3]. Kanda et al. [4] reported 3 cases of sudden sensorineural health loss (SSNHL) after treatment with INF and followed this report up with a study in 73 patients who received INF. In the study, auditory disorders (tinnitus, hearing loss) were seen in 43.8% of patients and SSNHL was seen in 36.9% of cases. The authors concluded that hearing disorders often occur in the later stages of treatment with INF and will improve 7 to 14 days after stopping the treatment. The cause of hearing loss was considered to be autoimmune. Other case reports include a group of 6 patients with hepatitis C who were receiving pegylated-INF-*α* and ribavirin and had hearing loss in either one or both ears [5]. In all of the cases, hearing loss did not resolve completely after stopping treatment with INF-*α*. Similar observations have been reported in 3 other case studies [2, 6, 7]. Patients receiving either INF-*α* or pegylated-INF-*α* because of chronic hepatitis B or C suffered SSNHL and tinnitus in one or both ears and in one case gait disorder as well, following 2 to 6 months of treatment. 

In addition to the prospective study by Kanda et al., the only other study of a cohort of patients was conducted by Görür et al. in 2001 [8]. In the study, 27 patients with chronic hepatitis B received treatment with INF-*α* and had their auditory threshold at 7 frequencies (250–8000 Hz) measured before treatment and after 1, 7, 21, and 28 days of treatment. In 9 patients, hearing loss was first detected after 7 days of treatment and the degree of hearing loss increased to a maximum at 21 days but did not exceed 20 dB in any frequency. Hearing loss was improved at one month following cessation of treatment. The authors concluded that INF-*α* treatment in patients with hepatitis B may cause mild, reversible SNHL.

In view of the limited information available on hearing disorders associated with treatment with INF-*α*, the purpose of this study was to (1) determine the rate of hearing disorders (tinnitus, hearing loss) in patients with chronic hepatitis who received INF-*α*; (2) evaluate the level of hearing loss after one month of INF-*α* treatment; (3) assess the age distribution of hearing disorders due to INF-*α* usage; and (4) assess the association of gender with hearing disorders due to INF-*α* usage.

## 2. Materials and Methods

 In a before-after study, 24 patients with hepatitis B or C who underwent treatment with INF-*α* and 30 normal age and sex matched persons were included. The patients were referred from gastrointestinal and infectious disease clinics and specialists. In this study, audiometric and otoacoustic emission (OAE) tests were performed on the study and control participants before treatment (baseline) and after one week and one month of treatment. Hearing loss was defined as a greater than 10 dB decrease at each tested frequency compared with baseline. The results of the OAE tests were also compared with baseline. 

In the audiometric clinic, we took a history regarding previous hearing loss and other audiometric disorders, tinnitus and vertigo. Then a physical examination and inspection of the ears of  the patients was performed, followed by audiometric tests including measurements of speech reception threshold (SRT), speech discrimination score (SDS), pure-tone average (PTA), tympanography, and acoustic reflex. Finally, patients underwent OAE testing and transient-evoked OAE testing (TEOAE, Echoport ILO 288, Otodynamic Company, UK). These tests were repeated one week later and at the end of one month of treatment. A total of  88.8% of these patients were treated with pegylated-INF-*α* and the others were treated with conventional INF-*α*. At the end of the study period, we used SPSS software to describe and analyze the study results. Statistical significance was ascertained using the paired *t*-test in most cases, as well as the Fisher test and logistic regression. A *P* value of less than 0.05 was considered significant.

The study protocol was approved by our local research ethics committee and the conduct of the acoustic tests did not cause any problems or complications for the patients. Each of the study participants gave a written informed consent.

## 3. Results

### 3.1. Patient Characteristics and Occurrence of Hearing Disorders

The study included 18 patients (75%) who were being treated for chronic hepatitis C, with the remaining 6 patients being treated for chronic hepatitis B. Controls were 30 cases of a normal person who were selected randomly from those who referred to the clinic as an associated to the patients for audiologic evaluation without any history of related diseases. 

The female/male ratio and mean age between cases and controls were 6/18; 8/22 and 40 ± 11.45;  38 ± 12.67, respectively. Two groups did not show significant differences regarding sex and age. A total of 21 patients (87.5%) had no otologic disorders (otorrhea, hearing loss) before treatment, and only 3 (12.5%) had a positive previous history of otologic problems. Out of these 3 patients, 2 had conductive hearing loss because of perforation of the tympanic membrane. Examination of both ears in each patient before and after one month of treatment with interferon indicated that, with the exception of the two patients with perforation of the tympanic membrane in one ear, the remaining 46 (95.8%) ears were intact. 

One month after starting treatment, 9 patients out of 24 (37.5%) had some degree of hearing loss in one or both ears, while the other patients and all of the controls reported no problems. This increase in the number of patients with hearing problems was statistically significant compared with baseline (*P* = 0.022) and controls. All of the 9 patients who had hearing loss were also males, which was also statistically significant (*P* = 0.037). Among those with hearing problem (9 cases), 3 and 6 had hepatitis B and C, respectively, which did not show significant differences (*P* = 0.113). The mean age of all the patients was 40 years (range: 19–78 years) and the mean age of the people who had hearing loss was 51.6 years. The mean age of the people who did not suffer from hearing loss was 33.1 years, which was significantly younger in comparison with those who did have hearing loss (*P* = 0.002). Only 3 patients out of 24 (12.5%) complained of tinnitus and vertigo after one month of treatment. These patients had not had either symptom before treatment, but this result was not significant (*P* = 0.083).

### 3.2. Acoustic Reflex, Audiometry, and Otoacoustic Emission Results

 For the acoustic reflex test, 46 ears of 48 (95.8%) responded before and after treatment, with the remaining 2 ears being those with perforated tympanic membranes. All controls had normal reflexes.

The patients' and controls' audiometric results are illustrated in Tables 1 and 2. A comparison of the patients' mean SRT results before and after treatment showed that the mean SRT before treatment was 10.3 ± 0.8, and after one month of treatment it was significantly greater at 11.6 ± 1.1  (*P* = 0.009). There was no significant difference in mean SRT after one week of treatment. For bone conductance threshold measured by PTA before and after treatment, there was only a significant difference in mean threshold at 4000 Hz and 8000 Hz (Table 1). Among controls, the SRT results after one month did not show significant changes (10.1 ± 1.1 and 9.9 ± 1.3); pure tone audiometry results are listed in Table 2.

A comparison of the results of the OAE test before and after one month of treatment with INF-*α* showed a significant decrease in the number of patients who passed the test (*P* = 0.007). Before treatment, 93.7% of patients passed the OAE test and only 79.1% passed after one month of treatment. After one week of treatment, 89.6% of patients passed the test, but this was not significantly different from baseline (*P* = 0.159). According to TEOAE at each frequency tested, there was a progressive decrease in OAE amplitude after one week and one month of treatment. At 1 kHz, this decrease was seen in 41.7% of patients, at 1.4 kHz it was observed in 27.1% of patients, at 2 kHz and 2.8 kHz it was observed in 18.8% of patients, and at 4 kHz it was observed in 43.8% of patients. The average stimulus stability was 99.1 ± 0.10% and the mean reproducibility of the tests was 71.1 ± 3.7%, which demonstrates the reliability of the tests.

## 4. Discussion 

 In this study, we evaluated the effects of treatment with INF-*α* on hearing in 24 patients with hepatitis B or C. The patients were assessed before treatment and after one week and one month of treatment. We observed that hearing loss occurred in 37.5% of patients after one month of treatment. This result is similar to that in the Kanda et al. study, [4] which reported that hearing loss occurs in the later stages of treatment with INF-*α*. In contrast, in the study by Görür et al., [8] hearing loss was seen in 30% of cases on the 7th day of treatment, and the degree of hearing loss increased until the 21st day, but the impairment observed was not more than 20 dB at any frequency. The authors concluded that the use of INF-*α* in patients with hepatitis B can lead to mild, reversible SSNHL, but this result has not been fully confirmed in other studies. Although we saw some hearing loss after 7 days of treatment and 37.5% of our patients complained of hearing loss at the end of the first month, some of the study participants had hearing loss in some frequencies of more than 20 dB. In addition to the two studies just discussed, the other studies in this field are case reports. In these case reports, hearing loss generally occurred in the later stages of treatment and mostly occurred at high frequencies and at more than 20 dB, which is similar to our study. 

We also observed significant changes in the bone conduction threshold in the 4 kHz and 8 kHz frequencies after one month of treatment compared with baseline but not in any frequencies below 4 kHz. From this result, we can conclude that SSNHL in patients treated with INF-*α* can be observed after one week of treatment and it becomes significant at 4 kHz and higher frequencies after a month of treatment. Hearing impairment may develop in the lower frequencies with continued INF-*α* treatment.

The one aspect that differentiates our study from others is the confirmation of hearing loss in patients using OAE, which has not been done in previous studies. We chose TEOAE in this study because of its ease of use in the clinic and less distortion due to environmental noise in comparison with the distortion-product OAE (DPOAE) technique. Although DPOAE tests are more sensitive at high frequencies, TEOAE tests are more sensitive in low frequencies. DPOAE and TEOAE have similar sensitivities in the middle frequencies. We compared OAE results in patients before and after one month of treatment with INF and saw a significant difference, which confirmed the reported hearing loss. We specifically assessed patients for OAE threshold, and we saw a gradual decrease in OAE threshold after one week and one month of treatment. The greatest number of patients with a decrease in OAE threshold was observed at 4 kHz and then 1 kHz. We also saw these changes at 1.4 kHz, 2 kHz, and 2.8 kHz but in fewer patients.

This hearing loss at 4 kHz is similar to our audiometric findings, which did not show any significant changes at 1 kHz. These results verify the fact that OAE can detect hearing loss earlier than conventional audiogram and that it has an important predictive role at low frequencies.

This study also aimed to assess the effects of age and gender on hearing loss due to treatment with INF-*α*. Our results show that there is a significant relationship between hearing loss, male gender, and older age. This is somewhat different to previous studies. In their 1990 study, Kennedy et al. [9] evaluated the ototoxic effects of carboplatin in 27 patients using audiometry. The maximum hearing loss they detected was at 8000 Hz, but, in 2 cases, they saw hearing loss at 1000 Hz. The patients with hearing loss were older but unlike our study gender had no effect. In another study by Waltzman and Cooper [10], the incidence and pattern of metronidazole ototoxicity was evaluated in 21 patients, but the researchers did not find any causative relationship between the degree of hearing loss, age, gender, and previous hearing loss. Finally, a study in 1983 by D'Alonzo and Cantor [11] suggested that impaired renal function, pregnancy, concurrent use of another ototoxic drug, genetic susceptibility to ototoxicity, and noise-induced hearing loss can enhance the risk of ototoxicity risk. Other factors that were related to ototoxicity included age, gender, and previous hearing loss, but there was little reliable evidence of this in the study.

## 5. Conclusion

Our study indicates that there is a risk of hearing loss associated with treatment of chronic hepatitis with INF-*α*. The impairment can initially be detected in the higher frequencies but may also progress to the lower frequencies. The use of OAE may detect these changes earlier than a conventional audiogram. In addition, there is an association between gender and age and hearing loss as a result of treatment with INF-*α*. We suggest that all patients with hepatitis B or C undergo an audiogram before starting treatment with INF-*α* and that regular audiograms and, if possible, OAE tests should be performed during treatment. Patients should also be informed of the potential complication of hearing loss associated with the use of INF-*α* and instructed to consult a physician in the case of any hearing or vestibular symptoms during treatment. 

## Figures and Tables

**Table 1:** Bone conductance threshold measured by pure tone audiometry in cases.

Frequency (Hz)	Mean threshold		*P* value
	Before treatment (SD)	One month after treatment (SD)	
250	9.7 (0.9)	9.6 (1.0)	0.83
500	7.5 (0.7)	8.3 (0.8)	0.103
1000	7.4 (0.9)	8.3 (1.2)	0.088
2000	6.35 (0.9)	7.8 (1.5)	0.90
4000	9.38 (1.0)	17.9 (2.6)	0.001
8000	10.7 (1.2)	17.6 (2.6)	0.001

SD: standard deviation.

**Table 2:** Bone conductance threshold measured by pure tone audiometry in controls.

Frequency (Hz)	Mean threshold		*P* value
	Before treatment (SD)	One month after treatment (SD)	
250	8.9 (1.1)	9.3 (1.0)	0.94
500	8.5 (0.9)	8.9 (0.7)	0.87
1000	8.7 (0.7)	8.1 (0.3)	0.48
2000	9.1 (0.8)	8.8 (0.9)	1.10
4000	8.9 (0.9)	10.1 (1.1)	0.23
8000	11.2 (1.4)	10.9 (1.8)	0.89

SD: standard deviation.
